# Boosting Power Density of Microbial Fuel Cells with 3D Nitrogen‐Doped Graphene Aerogel Electrode

**DOI:** 10.1002/advs.201600097

**Published:** 2016-04-15

**Authors:** Yang Yang, Tianyu Liu, Xun Zhu, Feng Zhang, Dingding Ye, Qiang Liao, Yat Li

**Affiliations:** ^1^ Key Laboratory of Low‐Grade Energy Utilization Technologies and SystemsInstitute of Engineering ThermophysicsChongqing UniversityChongqing400030P.R. China; ^2^ Department of Chemistry and BiochemistryUniversity of California ‐ Santa CruzSanta CruzCA95064USA; ^3^ Key Laboratory for Advanced Technology in Environmental Protection of Jiangsu ProvinceYancheng Institute of TechnologyYancheng224051P.R. China

**Keywords:** microbial fuel cells, nitrogen‐doped graphene aerogels, power density, *Shewanella oneidensis*

## Abstract

A 3D nitrogen‐doped graphene aerogel (N‐GA) as an anode material for microbial fuel cells (MFCs) is reported. Electron microscopy images reveal that the N‐GA possesses hierarchical porous structure that allows efficient diffusion of both bacterial cells and electron mediators in the interior space of 3D electrode, and thus, the colonization of bacterial communities. Electrochemical impedance spectroscopic measurements further show that nitrogen doping considerably reduces the charge transfer resistance and internal resistance of GA, which helps to enhance the MFC power density. Importantly, the dual‐chamber milliliter‐scale MFC with N‐GA anode yields an outstanding volumetric power density of 225 ± 12 W m^−3^ normalized to the total volume of the anodic chamber (750 ± 40 W m^−3^ normalized to the volume of the anode). These power densities are the highest values report for milliliter‐scale MFCs with similar chamber size (25 mL) under the similar measurement conditions. The 3D N‐GA electrode shows great promise for improving the power generation of MFC devices.

## 1 Introduction

Treatment of municipal wastewater is an energy‐intensive process, which needs an average energy density of ≈0.6 kW h to treat 1 m^3^ wastewater.^1^ This large energy consumption can be potentially compensated by extracting energy from wastewater, which contains an average energy density of ≈1.9 kW h m^−3^ stored as chemical energies in the form of various organic compounds.^1^ Microbial fuel cells (MFCs) are devices that can recover and transform these chemical energies to bioelectricity via bio‐oxidation with the help of electrogenic bacteria (e.g., *Shewanella oneidensis* and *Escherichia coli*).^2^ However, the relatively low power density of MFCs (typically in the order of several W m^−3^ for milliliter‐scale device)^3^ and the high capital cost associated with fabrication and operation processes severely hinders their potentials for practical applications. A major barrier of increasing power density is the poor interaction between bacteria and electrode,^4^ due to limited surface area of bioanode and the existence of inaccessible interior space for bacteria and electron mediators (e.g., extracellular polymers and electron shuttles).^5^ Design and fabrication of bioanodes with large surface area and accessible interior surface are critical for improving the power density of MFCs.

3D carbon‐based electrodes are promising candidates for addressing the aforementioned challenges.^6^ In general, carbon‐based materials are electrically conductive and chemically stable, which satisfy two basic requirements for MFC bioanodes. In addition, 3D morphology with large porosity provides easy access for bacteria as well as culture medium to the electrode interior space, and thus, increases the amount of bacterial colonies per unit volume. To date, many commercial 3D carbon‐based materials, such as graphite brush^7^ and carbon fiber nonwovens^8^ have been investigated as bioanodes. Under the same testing conditions, the MFCs with 3D carbon electrodes showed improved power densities compared to MFCs with plain carbon paper electrodes. In addition to these commercially available 3D carbon electrodes, a number of new 3D bioanodes have been reported. Xie *et al*. developed MFCs with carbon nanotubes (CNTs)–textile^9^ and CNTs–sponge^10^ as bioanodes. The MFCs with CNTs–textile and CNTs–sponge bioanode achieved high areal power densities of 1098 and 1570 mW m^−2^ (normalized to the geometric surface area of anodes), respectively. Based on the success of CNT‐based electrode, a variety of 3D graphene‐based materials, such as chitosan/vacuum‐stripped graphene scaffold,^5^ polyaniline (PANI)/graphene‐coated nickel foam,^4^ graphene–sponge^11^ and graphene‐coated nickel foam^12^ have also been demonstrated as bioanodes. They achieved the maximum areal power density of 1530 mW m^−2^ (normalized to the geometric surface area of the bioanode)^5^ and volumetric power density of 661 W m^−3^ (normalized to the volume of the bioanode).^12^ However, we believe that there is still room to further improve the volumetric power density of MFCs by enhancing the accessible surface area of 3D electrodes. Recently, graphene aerogels have received increasing attention as an electrode material for electrochemical devices. Graphene aerogel is a form of graphene monolith with 3D networks built up with many randomly oriented graphene sheets.^13^ It possess much higher surface area (≈100–500 m^2^ g^−1^) than other carbon materials (typically <10 m^2^ g^−1^) such as carbon cloths, carbon black powder, graphite powder as well as some porous metal substrates, e.g., nickel foams (≈0.1 m^2^ g^−1^). Because of the aforementioned advantages, graphene aerogels have already been demonstrated as promising electrode materials for supercapacitors,^14^ lithium‐ion batteries,^15^ and oxygen reduction reactions.^16^ However, the use of graphene aerogels as electrodes of MFCs is rare. Qiao *et al*. demonstrated an MFC with l‐cysteine tailored porous graphene aerogel as bioanode.^17^ The MFC delivered a large areal power density of 679.7 mW m^−2^ (normalized to the geometric surface of the bioanode, 1 cm^2^) but limited volumetric power density of 0.68 W m^−3^ (normalized to the volume of the anodic chamber, 100 mL).

In this work, we demonstrated the first time the MFC with porous nitrogen‐doped graphene aerogel (denoted as N‐GA) as bioanode. The MFC achieved a low internal resistance of 55.4 Ω and a remarkably high volumetric power density of 225 ± 12 W m^−3^ based on the volume of the anode chamber (equivalent to 750 ± 40 W m^−3^ normalized to the volume of the N‐GA), which is substantially higher than those of MFCs with conventional carbon cloth (denoted as CC) bioanode (16.8 ± 0.6 W m^−3^, normalized to the volume of the anode chamber) as well as reduced graphene oxide coated nickel foam (denoted as rGO‐Ni) bioanode (82.8 ± 1.3 W m^−3^, normalized to the volume of the anode chamber)^12^ under the similar measurement conditions. The power density is also the highest among all milliliter‐scale MFCs with similar chamber size (≈25 mL) and the same catholyte (potassium ferricyanide). The excellent performance can be ascribed to two factors. First, the 3D macroporous structure of the N‐GA promotes bacterial colonization on both the exterior and interior surface of the electrode to enhance the utilization efficiency of bioanodes.^7^, ^18^ Second, the enhanced electrical conductivity as a result of nitrogen‐doping can facilitate transport of electrons generated by bacteria to external circuit, and thus, improve the power density.

## 2 Results and Discussions

### 2.1 Physical Properties of N‐GA

**Figure 1:**
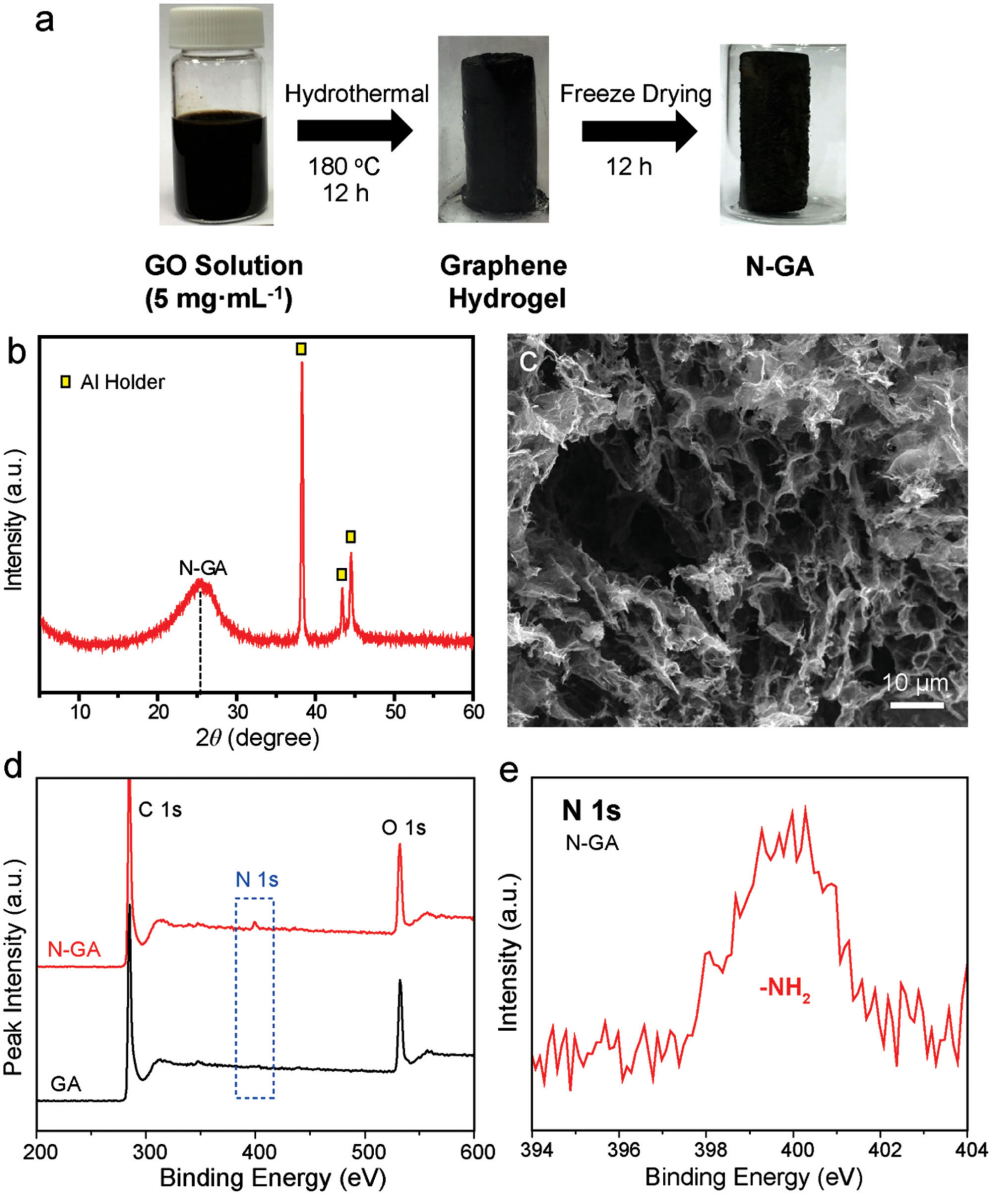
a) Schematic illustration showing the synthetic procedure of N‐GA. b) XRD pattern and c) SEM image of N‐GA. d) XPS survey spectra of N‐GA and GA. e) High‐resolution N 1s XPS spectrum of N‐GA.

**Figure** **1**a illustrates the key steps of synthesizing N‐GA (Experimental Section). The nitrogen‐doped graphene hydrogels were first prepared from GO precursor solution via a hydrothermal reaction and were subsequently freeze‐dried to obtain the N‐GAs. In the XRD measurement (Figure 1b), N‐GA exhibits a broad diffraction peak centered at 25°, which is the characteristic peak of graphene aerogel structure.^13^ SEM image (Figure 1c) revealed that the N‐GA possessed 3D interconnected porous structure with macro‐pores between 5 and 10 μm in diameter. The size of these macropores is large enough to allow diffusion of bacteria (*Shewanella oneidensis*)‐enriched solution into the interior portion of the 3D network, which can increase the surface area available for bacterial colonization.^19^ Figure 1d compares the XPS survey spectra of GA and N‐GA. The spectrum of N‐GA showed a small peak at ≈ 400 eV, which can be assigned to N 1s, while no peak could be observed in the spectrum of GA at the same binding energy. The atomic percentage of N in N‐GA was determined to be 2.24% (Table S1, Supporting Information). The N 1s peak of N‐GA (Figure 1e) centered at 399.8 eV is consistent with the binding energy reported for amine groups in nitrogen‐doped graphene.^20^ Besides, the Fourier‐transformed infrared spectrum of N‐GA (Figure S1, Supporting Information) also proved the existence of amine groups. It is believed that the amine groups are produced from the reaction between ammonia and epoxide groups or carboxyl groups on the GO sheets.^20^

**Figure 2:**
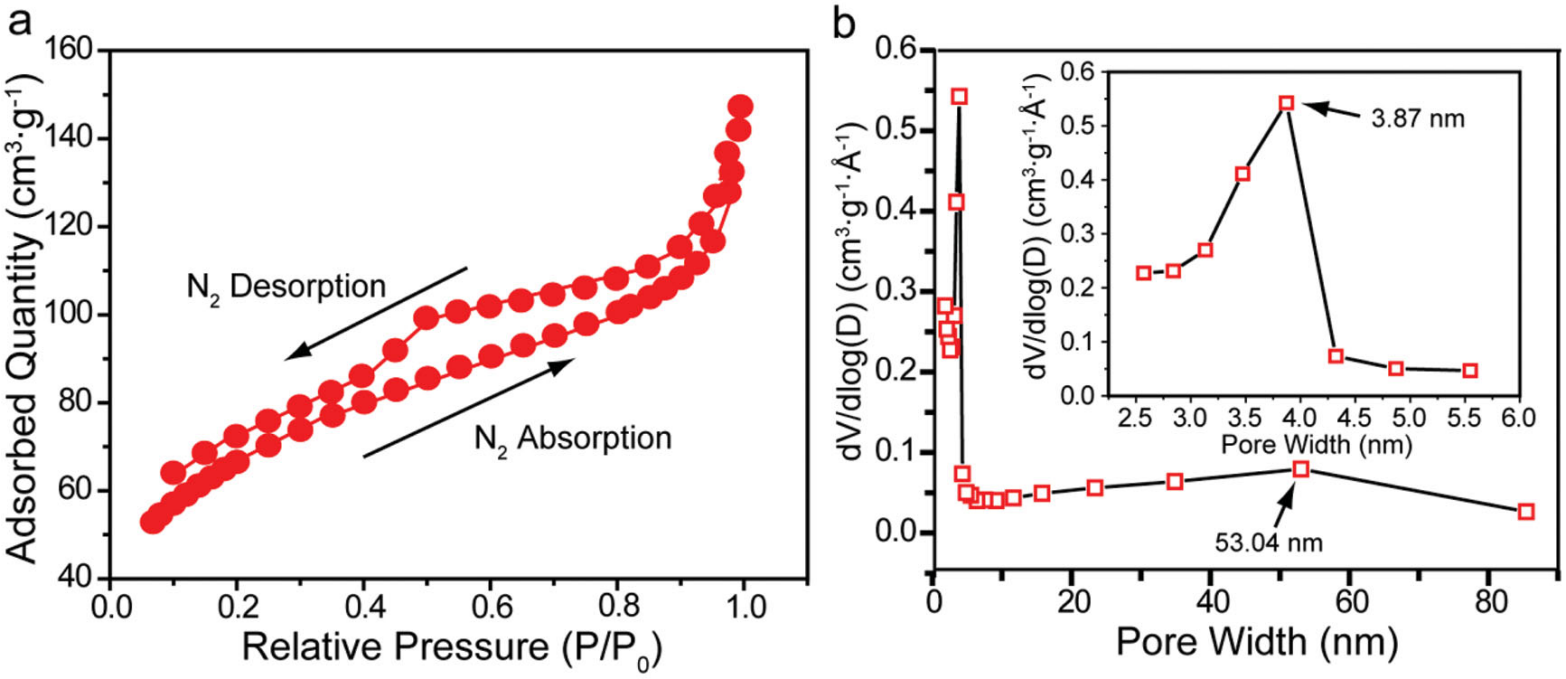
a) Nitrogen adsorption/desorption isotherm collected at 77 K for N‐GA. b) Pore size distribution of N‐GA. Inset shows the pore size distribution of micropores.

Brunauer‐Emmett‐Teller (BET) test was carried out to further characterize the porous structure of N‐GA. As shown in **Figure** **2**a, N‐GA exhibits a type‐IV isotherm that is a characteristic profile of mesoporous structures.^21^ The pore size distribution curve indicates that the majority of pores lies in the width ranging from 1.5 to 5 nm, proving the existence of both mesopores and micropores (Figure 2b). There is also a broad peak centered at 53 nm corresponding to macropores, in consistent with the SEM image of N‐GA (Figure 1c). The results proved that N‐GA had a hierarchical porous structure. The BET surface area of the N‐GA was determined to be 235.95 m^2^ g^−1^, which was substantially larger than that of CC (5.3 m^2^ g^−1^)^22^ and rGO‐Ni (46.76 m^2^ g^−1^, Figure S2, Supporting Information). Moreover, we also determined the electrochemically accessible surface area (ECSA) of CC, rGO‐Ni, and N‐GA (Figure S3, Supporting Information). Again, the N‐GA possesses the highest ECSA among the three electrodes, consistent with the BET results. The large surface area and hierarchical porous structure containing micro‐, meso‐, and macropores offer large accessible surface for colonization of bacteria and interaction with electron mediators.

### 2.2 Bioelectricity Generation

**Figure 3:**
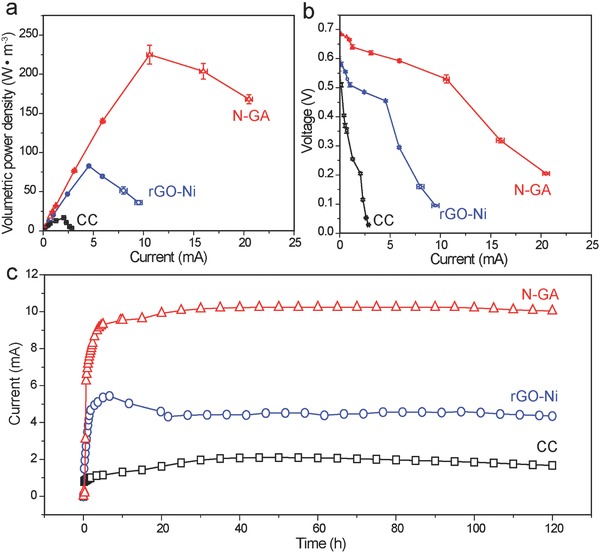
a) Volumetric power density and b) polarization curves collected for the three MFCs. The power density was calculated based on the volume of anodic chamber (25 mL). The error bars represent the standard deviation evaluated based on data collected in triplicate. c) Amperometric *I*–*t* curves collected for the three MFCs operated at maximum power density. The external resistor connected in series with the CC MFC, rGO‐Ni MFC, and N‐GA MFC are 100, 100, and 50 Ω, respectively.

To evaluate the performance of N‐GA, we fabricated an MFC using the N‐GA as anode (denoted as N‐GA MFC). The performance of MFCs using the CC and rGO‐Ni as anodes (denoted as CC MFC and rGO‐Ni MFC, respectively) was also tested for comparison. Notably, as shown in **Figure** **3**a, the N‐GA MFC displayed an outstanding maximum volumetric power density of 225 ± 12 W m^−3^ normalized to the total volume of the anodic chamber (equivalent to a volumetric power density of 750 ± 40 W m^−3^ normalized to the volume of the anode), which is higher than the CC MFC (17 ± 1 W m^−3^) and rGO‐Ni MFC (83 ± 2 W m^−3^). To our knowledge, these volumetric densities are among the highest values of all milliliter‐scale MFCs with similar chamber size and the same catholyte (potassium ferricyanide) (**Table** **1**). In addition, the maximum areal power density (normalized to the geometric surface area of N‐GA, 9.4 cm^2^) for N‐GA MFC is 1990.8 ± 106.1 mW m^−2^, which is much higher than the values of CC MFC (78.2 ± 2.7 mW m^−2^) and rGO‐Ni MFC (517.6 ± 8.0 mW m^−2^). Significantly, the maximum areal power density is also among the state‐of‐the‐art performance and considerably higher than other MFCs with carbon‐based bioanodes, including the MEMS MFC (47 mW m^−2^),^23^ carbon nanotube‐based MFC (830 ± 10 mW m^−2^)^24^ and graphene/PANI foam‐based MFC (768 mW m^−2^).^25^ From the polarization curves shown in Figure 3b, the N‐GA MFC also exhibited the highest open circuit potential (0.69 ± 0.01 V) with the largest maximum current (20.50 ± 0.35 mA) among the three MFCs. The MFC was able to deliver a stable current in at least 5 d (Figure 3c). We ascribe these excellent performances to two possible reasons: 1) the hierarchical porous structure not only offers large surface area, but also allows bacterial colonization on both exterior and interior surface of the graphene aerogel electrode, and 2) nitrogen doping reduces the internal resistance of N‐GA electrode.

**Table 1:** Performance of milliliter‐scale MFCs with carbon‐based bioanodes

Bio‐anode material	Open‐circuit potential [V]	Bacteria	Maximum volumetric power densitya) [W m^−3^]	Maximum volumetric power densityb) [W m^−3^]	Refs.
Plain graphite	–	Anaerobically activated sludge	0.4 (45 mL)	–	32
Carbon felt	≈0.56	Mixed bacteria	0.8 (36 mL)	10.1 (2.8 mL)	33
Polyaniline‐coated carbon felt	≈0.60	Mixed bacteria	1.1 (36 mL)	13.7 (2.8 mL)	33
Polyaniline/poly(1,8‐diaminonaphthalene)‐coated carbon felt	≈0.50	Mixed bacteria	93.3 (36 mL)	933 (3.6 mL)	34
Carbon cloth	≈0.70	Mixed bacteria	60.0 (28 mL)	–	35
Polyaniline/TiO_2_‐coated Ni foam	0.88	*E. coli*	135 (25 mL)	–	36
3D graphene scaffold	≈0.68	*S. oneidensis*	27 (25 mL)	661 (1.02 mL)	12
Carbon felt	0.17	Anaerobically activated sludge	10.8 (20 mL)	–	37
Carbon nanotube sponge	0.25	Anaerobically activated sludge	14.1 (20 mL)	940 (0.3 mL)	37
CC	0.58 ± 0.02	*S. oneidensis*	17 ± 1 (25 mL)	56 ± 2 (7.5 mL)	This Work
rGO‐Ni	0.60 ± 0.01	*S. oneidensis*	83 ± 2 (25 mL)	276 ± 4 (7.5 mL)	This Work
N‐GA	0.69 ± 0.01	*S. oneidensis*	225 ± 12 (25 mL)	750 ± 40 (7.5 mL)	This Work

^a)^Normalized to the total volume of the anodic chamber (listed in the parenthesis);

^b)^Normalized to the total volume of bioanode (listed in the parenthesis).

### 2.3 Bacterial Colonization

**Figure 4:**
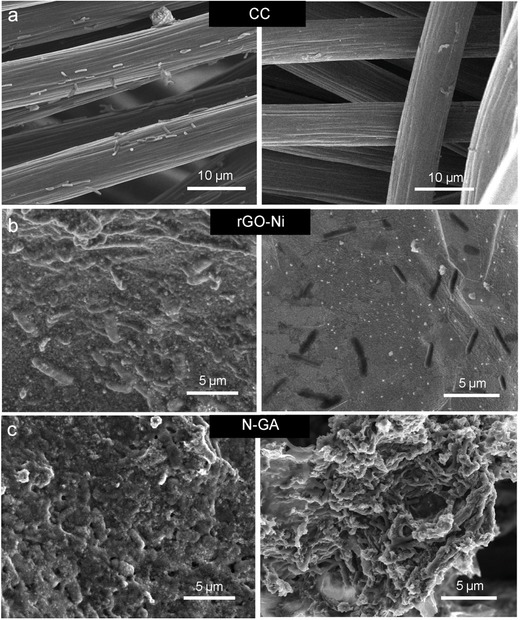
SEM images collected at the exterior surface (left column) and interior surface (right column) of *S. oneidensis* colonized a) CC, b) rGO‐Ni, and c) N‐GA electrode, respectively.

SEM images were collected for bacterial colonized CC, rGO‐Ni, and N‐GA electrodes after testing in MFC for 5 d. In the case of CC, bacterial communities were sparsely distributed on exterior surface of the electrode but hardly observed on the interior surface of CC fibers (**Figure** **4**a). Since carbon cloths are made of carbon fiber yarns, which are closely packed together and lack of mesopores,^22^ it makes them impenetrable for bacteria. On the contrary, bacterial colonies were clearly observed on both the exterior and interior parts of rGO‐Ni and N‐GA anodes (Figure 4b,c). As shown in right column of Figure 4c, a lot of bacterial cells are located in the interior surface of N‐GA (compared to bare N‐GA in Figure S4, Supporting Information). In contrast, there is very limited number of bacteria on the interior surface of rGO‐Ni. Specifically, we estimated the bacterial area density on the interior surface of rGO‐Ni and N‐GA based on the SEM images and the following equation:

$$\rho =10^{6}\times N/A$$

where *ρ* is the bacterial area density (cells mm^−2^), *N* is the number of bacterial cells shown in corresponding SEM images and *A*, the area shown in corresponding SEM images estimated using the scale bars (μm^2^). The bacterial areal density on the interior surface of rGO‐Ni and N‐GA is estimated to be 19 102 and 89 779 cells mm^−2^, respectively. It explains why the MFC with rGO‐Ni or N‐GA as anode achieved considerably higher current and power density than the CC MFC. The better performance of N‐GA MFC relative to rGO‐Ni MFC is believed to be due to two reasons. First, N‐GA has higher surface area than that of rGO‐Ni, providing more sites for bacterial colonization, and thus, enhances bioelectricity generation. Second, the nitrogen‐doping could increase the affinity between N‐GA electrode and bacteria. The improvement of bacterial colonization in the interior surface of N‐GA was ascribed to the formation of surface nitrogen‐containing functional groups (e.g., amine groups) that turned graphene sheets into positively charged.^18^, ^26^ The positive surface charge of N‐GA is supported by the zeta‐potential measurements (Figure S5, Supporting Information). As a result, bacterial cells with inherent negative charges are more favorable to adhere to the substrate due to the electrostatic attraction. The increased biofilm thickness of biofilm of N‐GA ensures the high power density of the N‐GA MFC, as demonstrated previously.^27^

### 2.4 Electrochemical Impedance Study

**Figure 5:**
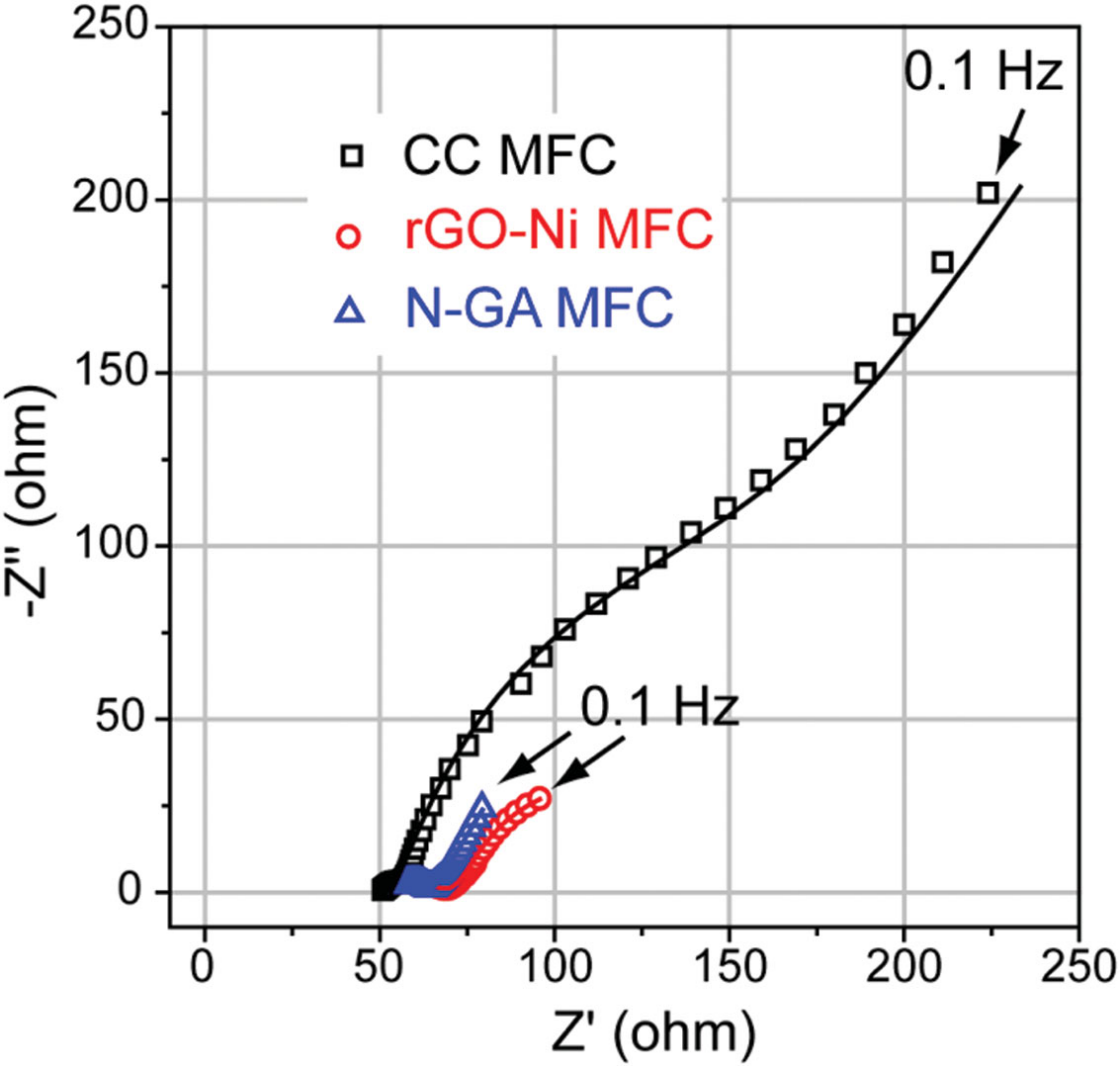
Nyquist plots of the three MFCs. Open dots and solid lines represent experimental data and fitting curves created by simulating the experimental data using the equivalent electric circuit (see Supporting Information), respectively.

To evaluate the effect of nitrogen doping on the electrical conductivity of GA and how it affects the performance of MFC, we conducted electrochemical impedance spectroscopy (EIS) measurements for the three MFCs (**Figure** **5**). By fitting the EIS data with the equivalent electric circuit shown in Figure S6 (Supporting Information), ohmic resistance (*R*_Ω_) and charge transfer resistance of anode (*R*_ct,a_) for the three MFCs were obtained (Table S2, Supporting Information). The rGO‐Ni MFC has the lowest *R*_Ω_ value due to the presence of highly conductive nickel frameworks (electrical conductivity of nickel: ≈10^7^ S m^−1^), while the other two MFCs have comparable *R*_Ω_ values. Notably, the N‐GA MFC has the smallest *R*_ct,a_ (11.05 Ω), which was 67 times and 4.5 times lower than that of CC MFC (748 Ω) and rGO‐Ni MFC (61.45 Ω), respectively. The charge transfer resistance is mainly due to the resistance of extracellular electron mediator diffusion.^28^ The smallest *R*_ct,a_ value suggests that the diffusion of electron mediators in N‐GA is most efficient among the three anodes. The results also explain why N‐GA MFC achieved the highest power density since the diffusion of electron mediators is one of the key factors determining power density. In addition to *R*_Ω_ and *R*_ct,a_, diffusion resistance (*R*_D_) that mainly accounts for the resistance of bacterial culture diffusion has been also widely used in evaluating the performance of MFCs.^29^ As listed in Table S2 (Supporting Information), N‐GA MFC exhibits a *R*_D_ of 81.14 Ω, which is smaller than that of rGO‐Ni MFC (99.52 Ω) and substantially lower than that of CC MFC (237.1 Ω). This result supports our hypothesis that the porous structure of N‐GA allows efficient diffusion of bacterial culture.

Moreover, we also investigated the effect of nitrogen‐doping on internal resistance of MFCs. Previous studies have showed that nitrogen‐doping can substantially increase the electrical conductivity of graphene by lowering the energy gap between the highest occupied molecular orbital and lowest unoccupied molecular orbital.^30^ *I*–*V* curve and cyclic voltammograms collected for graphene aerogel without N‐doping (GA) and N‐GA (Figure S7, Supporting Information) also confirmed the enhanced electrical conductivity of N‐GA. As a result, the N‐GA MFC displayed a considerably smaller *R*_Ω_ (55.4 Ω) than that of GA (96.2 Ω) (Figure S8, Supporting Information), which is beneficial for N‐GA MFC to achieve the high power density.

## 3 Conclusion

In this work, we have synthesized the highly conductive and porous nitrogen‐doped graphene aerogel MFCs. The N‐GA MFC achieved an outstanding volumetric power density of 225 ± 12 W m^−3^ with a high open‐circuit potential of 0.69 ± 0.01 V. The incorporation of N‐GA bioanode makes our MFC achieved the highest volumetric power density ever reported for milliliter‐scale MFCs under similar testing conditions. This work demonstrates the key factor to improve power density of MFCs is twofolded, the synthesis of 3D electrodes with large surface area as well as the successful utilization of interior surface of substrates. Large surface area is important not only for anode but also cathode, so we believe that the N‐GA could also be a good cathode material (Figure S9, Supporting Information). Besides, the facile hydrothermal preparation of N‐GAs can be easily scaled up, and thus opens up tremendous opportunities to further push the power densities of large‐scale MFCs.

## 4 Experimental Section

*Preparation of Graphene Oxide Solution*: GO solution was prepared via a modified Hummer's method reported elsewhere^31^ and was used as the precursor for N‐GA. 2.5 g graphite powders were mixed with 23 mL concentrated sulfuric acid (98 wt%) and 10 mL concentrated nitric acid (65 wt%) in a glass beaker at 0 °C. Then 3 g potassium permanganate was slowly added to the mixture at 35 °C, stirred for 3 h and subsequently diluted by adding 40 mL deionized water. After stirring for 12 h, the solution was further diluted by adding 200 mL deionized water. Then, hydrogen peroxide aqueous solution (30 vol%) was added dropwise until the color of the mixture turned from brown to gold. Once the solution temperature drop to room temperature, the solution was centrifuged at 1500 rpm for 30 min and the precipitation was collected and washed with ample amount of deionized water until the pH value was closed to 7. Finally, the precipitation was redispersed in deionized water to form a GO solution with a concentration of 5 mg mL^−1^.

*Synthesis of N‐GA*: N‐GAs were prepared by a facile hydrothermal method reported elsewhere.^16^ 8 mL of concentrated ammonium hydroxide solution was mixed with 20 mL of the 5 mg mL^−1^ GO precursor solution. Then, the mixture was transferred into a Teflon‐lined reactor and sealed in a steel autoclave. The autoclave was heated in an electric oven at 180 °C for 12 h. The nitrogen‐doped graphene hydrogel was formed in the reactor. The graphene hydrogel was immersed in deionized water overnight to remove impurities. Finally, the N‐GA was obtained by freeze‐drying the graphene hydrogel for 12 h. For comparison, graphene aerogels without N‐doping (denoted as GA) were prepared using the same method except concentrated ammonium hydroxide solution was replaced by the same amount of de‐ionized water for the hydrothermal reaction.

*Material Characterizations*: The morphology of the N‐GA was characterized by a field‐emission scanning electron microscope (SEM, Hitachi S‐4800 II). The specific surface area of N‐GA was determined by Brunauer–Emmett–Teller (BET) method through nitrogen adsorption–desorption at 77 K. Pore size distribution was examined by a porosimetry analyzer (Micromeritics, ASAP 2020, USA) and analyzed according to the Barett–Joyner–Halenda (BJH) model. The composition of N‐GA was characterized by X‐ray diffraction (XRD, Rigaku Americas Miniflex Plus powder diffractometer) and X‐ray photoelectron spectroscopy (XPS, ESCALAB 250 Xi XPS System, Al K*α* radiation).The binding energies of XPS peaks were calibrated using the C 1s photoelectron peak at 284.6 eV as the reference. The chemical composition of the nitrogen‐containing functional groups was probed by Fourier‐transform infrared spectrometer (Spectrum One, Perkin‐Elmer, spectral resolution: 4 cm^−1^). Zeta‐potential measurements were conducted with a Nano ZS Zetasizer (model ZEN3600, Malvern Instruments) using a He‐Ne laser at a wavelength of 632.8 nm.

*MFC Setup*: A drop of *Shewanella oneidensis* MR‐1 (ATCC 700550) bacterial culture was inoculated into 50 mL of fresh trypticase soy broth (TSB, BD Biosciences) and incubated under aerobic conditions with continuous shaking at 30 °C for 12 h. Then the bacterial suspension was transferred into the anodic chamber and purged with nitrogen gas for 15 min to remove the dissolved oxygen gas in the solution. 20 mL of 15.6 g potassium ferricyanide (K_3_[Fe(CN)_6_]) diluted in 100 × 10^−3^ M phosphate buffer solution (PBS) was used as catholyte and injected into the cathodic chamber. The two chambers were separated by a piece of cation‐exchange membrane (CEM). The volume of each chamber was 25 mL. Carbon cloth (CC6 Plain, Fuel Cell Earth LLC) and N‐GA were used as cathode and bioanode, respectively. The MFC was then connected to an external resistance (500 Ω) and the voltage was recorded by a data acquisition unit (Keithley 2700). Once the voltage dropped to background, fresh TSB medium was injected to the anodic chamber. This process was repeated for three times to inoculate *Shewanella oneidensis* MR‐1 cells. As comparison, MFCs with a piece of carbon cloth (CC6 Plain, Fuel Cell Earth LLC) and a piece of reduced graphene oxide coated nickel foam (rGO‐Ni) as bioanodes were assembled according to the same aforementioned method. The volumes of N‐GA, CC and rGO‐Ni were all ≈7.5 cm^3^.

*Electrochemical Measurements*: Electrochemical impedance spectroscopy (EIS) was performed in a frequency range from 0.01 to 10000 Hz using a CHI 660D Electrochemical Workstation (CH Instrument). An external voltage of 0.1 V was applied to the MFCs with a perturbation of 10 mV during the test. EIS data were fitted using the *ZsimpWin* software. To collect the polarization curves, MFCs were connected in series with various external resistors (3900, 1000, 710, 510, 200, 100, 50, 20 Ω). The current (*I*) was evaluated according to Ohm's law:

(1) I = U/R

where *U* is the potential recorded by the data acquisition unit and *R* is the resistance of the external resistor. The volumetric power density (*P*_v_) was calculated using

(2) $$P_{v}\;=\;U\;\times \;I/V$$

where *V* is the volume of the bioanode or the anodic chamber. Amperometric *I*–*t* curves were collected in a fed batch model for 5 d at maximum power density. The external resistor connected in series with the CC MFC, rGO‐Ni MFC, and N‐GA MFC has a resistance of 100, 100, and 50 Ω, respectively. The potential was recorded by the data acquisition unit (Keithley 2700). The recorded potential was then converted to current according to the Ohm's law. Electrochemically accessible surface area of each electrode was determined by cyclic voltammetry using 5 × 10^−3^ m potassium ferricyanide aqueous solution containing 0.1 m LiClO_4_ as the supporting electrolyte.

## Supporting information

As a service to our authors and readers, this journal provides supporting information supplied by the authors. Such materials are peer reviewed and may be re‐organized for online delivery, but are not copy‐edited or typeset. Technical support issues arising from supporting information (other than missing files) should be addressed to the authors.

Supplementary
