# Supplementary material for: Boosting Power Density of Microbial Fuel Cells with 3D Nitrogen‐Doped Graphene Aerogel Electrode
Source: Adv Sci (Weinh). 2016 Apr 15;3(8):1600097. doi: 10.1002/advs.201600097 (PMC5074258; doi:10.1002/advs.201600097)
Supplement: Supplementary file 1 — Supplementary [file ADVS-3-0c-s001.pdf]

Copyright WILEY-VCH Verlag GmbH & Co. KGaA, 69469 Weinheim, Germany, 2016.

ADVANCED SCIENCE
Open Access

Supporting Information

for *Adv. Sci.,* DOI: 10.1002/advs.201600097

Boosting Power Density of Microbial Fuel Cells with 3D Nitrogen-Doped Graphene Aerogel Electrode

Yang Yang, Tianyu Liu, Xun Zhu, Feng Zhang, Dingding Ye, Qiang Liao, and Yat Li**

Copyright WILEY-VCH Verlag GmbH & Co. KGaA, 69469 Weinheim, Germany, 2013.

Supporting Information

Boosting Power Density of Microbial Fuel Cells with Three-dimensional Nitrogen-doped Graphene Aerogel Electrode

Yang Yang†[a,b], Tianyu Liu†[b], Xun Zhu[a], Feng Zhang[c], Dingding Ye[a], Qiang Liao[a], Yat Li[b]**

[a] Key Laboratory of Low-Grade Energy Utilization Technologies and Systems, Institute of Engineering Thermophysics, Chongqing University, Chongqing, China, 400030

[b] Department of Chemistry and Biochemistry, University of California – Santa Cruz, Santa Cruz, California, United States, 95064

[c] Key Laboratory for Advanced Technology in Environmental Protection of Jiangsu Province, Yancheng Institute of Technology, Yancheng, China, 224051

† These authors contributed equally to this work.

Supplementary Figures and Tables

Table S1. Element Contents of GA and N-GA

Sample	C Atomic %	O Atomic %	N Atomic %
GA	81.51	18.49	-
N-GA	81.61	16.14	2.24

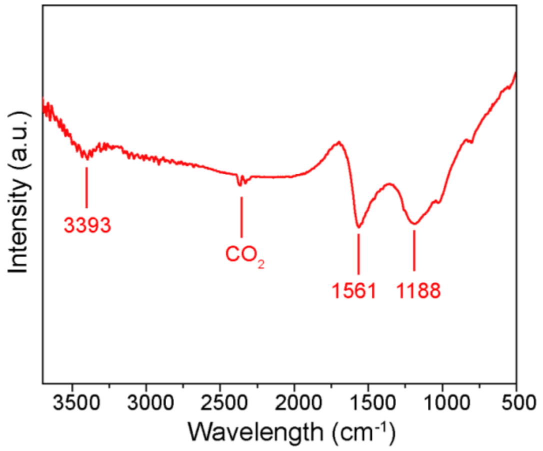

Figure S1. The FT-IR spectrum of N-GA. The characteristic peaks of the amine groups are labeled. The doublet at *ca.* 2300 cm^{-1} comes from carbon dioxide in the atmosphere.

As shown in Figure S1, the peak at 3393 cm^{-1}, 1561 cm^{-1} and 1188 cm^{-1} can be ascribed to

N-H stretching of amine groups, red-shifted in-plane vibration of C=C as a result of binding with amine groups, and C-N stretching, respectively.[1]

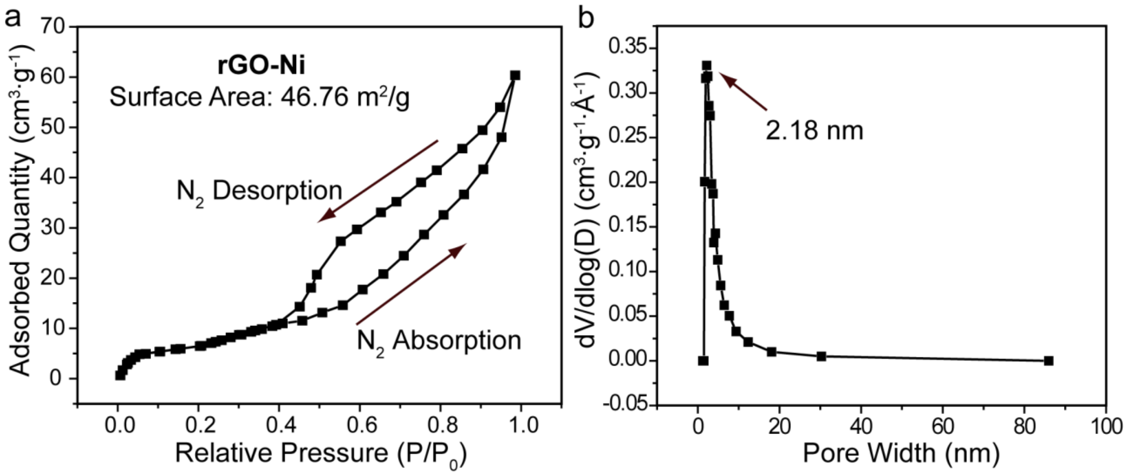

Figure S2. (a) Nitrogen adsorption/desorption isotherm and (b) pore size distribution of rGO-Ni.

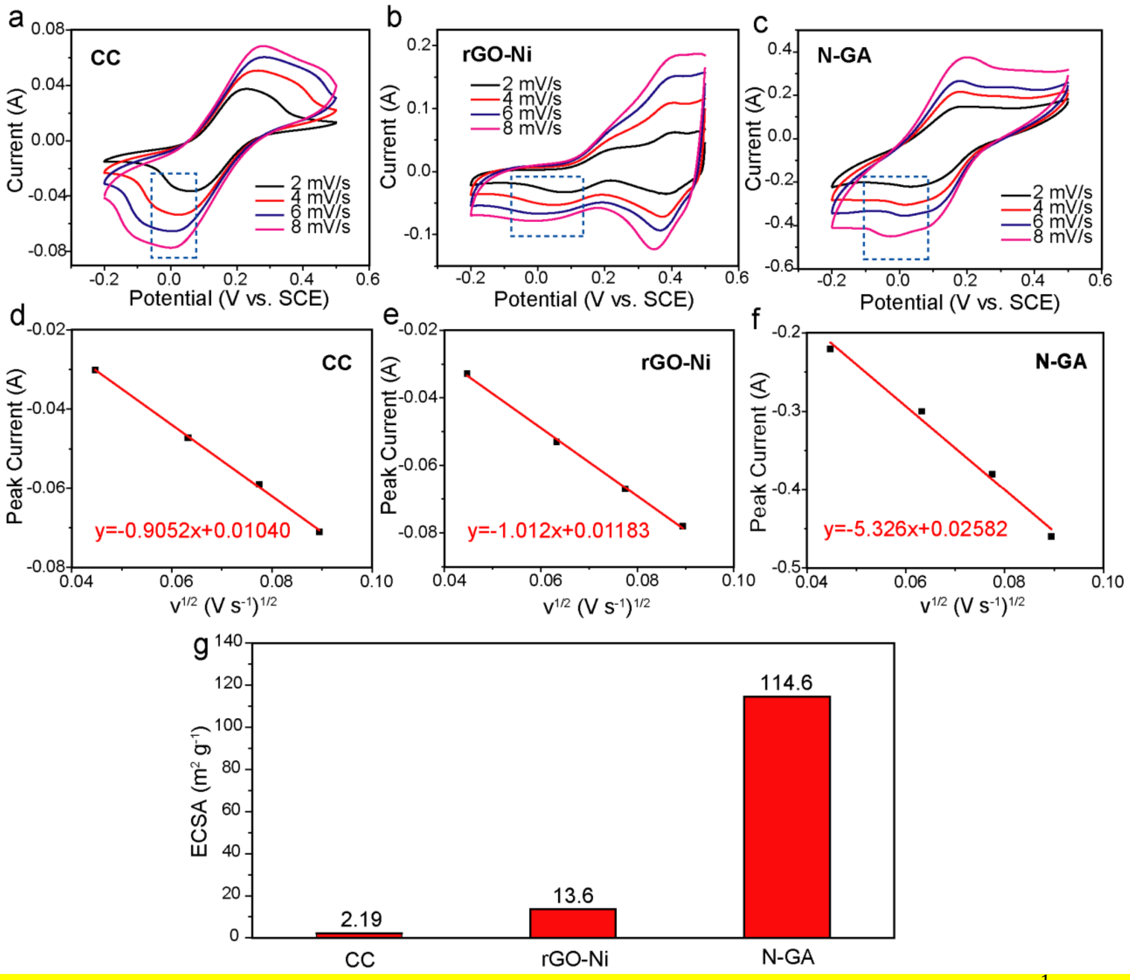

Figure S3. (a-c) CV diagrams collected at various small scan rates (2-8 mV s^{-1}) in 5 mM $K_3Fe(CN)_6$ containing 0.1 M $LiClO_4$ as supporting electrolyte. The dashed boxes highlight the cathodic peaks that are chosen to calculate ECSAs. (d-f) i_p vs. $v^{1/2}$ plots of the three bio-anodes. (g) Histogram compares the ECSA of the three bio-anodes.

To estimate the electrochemically accessible surface area (ECSA) of the bio-anodes, we have carried out the cyclic voltammetry (CV) in 5 mM potassium ferricyanide aqueous solution containing 0.1 M $LiClO_4$ as the supporting electrolyte. The ECSAs were evaluated using the following equation: [2]

$$i_p = (2.69\times10^5)n^{3/2}AD_0^{1/2}C_O^{*}v^{1/2} \quad \text{[Equation S1]}$$

where i_p (in A) stands for the peak current, n the number of electrons transferred in the following balanced equation (Equation S2, which we assumed 1 for this case), A (in cm^2) the ECSA (not normalized to total mass), D_0 the diffusion coefficient of $Fe(CN)_6^{3-}$ (0.7×10^{-5} cm^2 s^{-1} in aqueous solution[3]), C_o^* the bulk concentration of $Fe(CN)_6^{3-}$ (5×10^{-6} mol mL^{-1}), and v (in V s^{-1}) the scan rate. The ECSA can be evaluated from the slope of i_p vs. $v^{1/2}$ plot.

$$Fe(CN)_6^{3-} + e^- \rightarrow Fe(CN)_6^{4-} \quad \text{[Equation S2]}$$

To obtain the slope, CV curves of the three bio-anodes were collected at small scan rates (2-8 mV s^{-1}) (Figure S3a-c). The intensities (i_p) of the cathodic peaks obtained at *ca.* 0.02 V (vs. SCE) at various scan rates were measured (the anodic peaks at ca. 0.2 V *vs.* SCE should also work but since for the rGO case, the anodic peak merges into the redox peaks of rGO, we chose the cathodic ones to calculate.). We plot i_p vs. $v^{1/2}$ and fit the data points with a straight line, the slopes can be read from the corresponding algebraic equations (Figure S3d-f). The ECSAs then are evaluated using the Equation R3. Figure R2g summarizes the calculated ECSA for the three bio-anodes. Despite all ECSAs are smaller than the values obtained from BET measurement (due to the presence of intrinsic resistance, non-flat surface, non-accessible micro-pores and variation of C_O^* *etc.*), the N-GA still possesses the highest ECSA among the three electrodes, consistent with the trend revealed by BET and the highest power density achieved by the N-GA MFC.

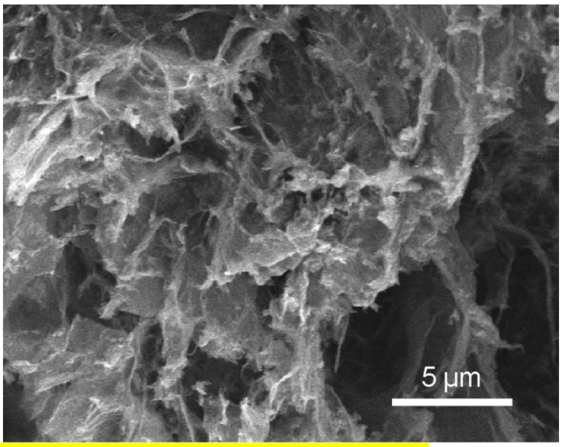

Figure S4. SEM images of the interior surface of bare N-GA.

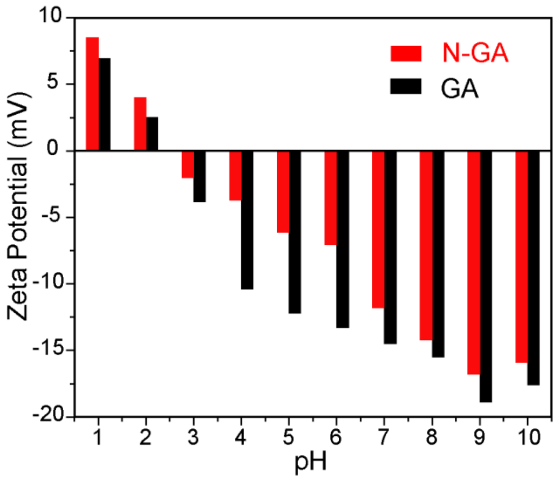

Figure S5. Zeta potentials of GA and N-GA aqueous dispersion collected at various pH values in water. 1 M HCl and 1 M NaOH aqueous solution were used to adjust pH value.

To show the amine groups imbibe positive charge, we measured the zeta-potentials of N-GA at various pH values and compared them with GA (Figure S5). Zeta-potential is a measurement on surface potential that largely depends on the surface charge.[4] The up-shifted zeta-potential of N-GA at pH=7 clearly revealed that surface of N-GA is more positive than that of GA, owing to the presence of positively charged amine groups, which is consistent with previous reports.[4-5]

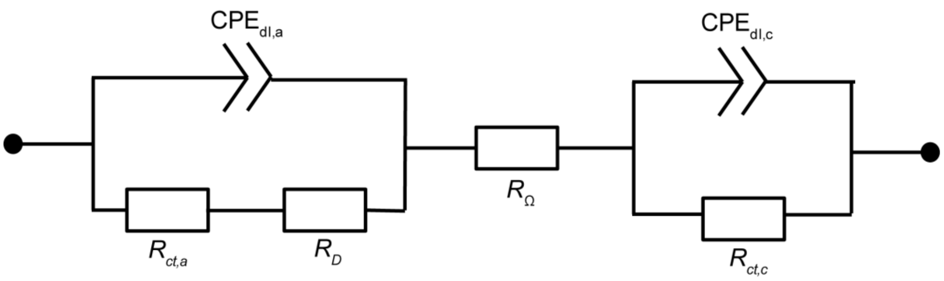

Figure S6. The equivalent circuit used for fitting EIS data. Abbreviations: R_Ω–ohmic resistance (including the resistance of membrane); CPE$_{dl,a}$ and CPE$_{dl,c}$–constant phase element associated to the double layer at the surface of anode and cathode, respectively; $R_{ct,a}$ and $R_{ct,c}$–charge transfer resistance of anode and cathode, respectively.

Table S2. Fitted parameters of some elements in the equivalent circuit

Anode	R_Ω/ohm	$R_{ct,a}$/ohm	R_D/ohm
CC	53.08	294.7	237.1
rGO-Ni	35.01	36.78	99.52
N-GA	54.01	9.32	81.14

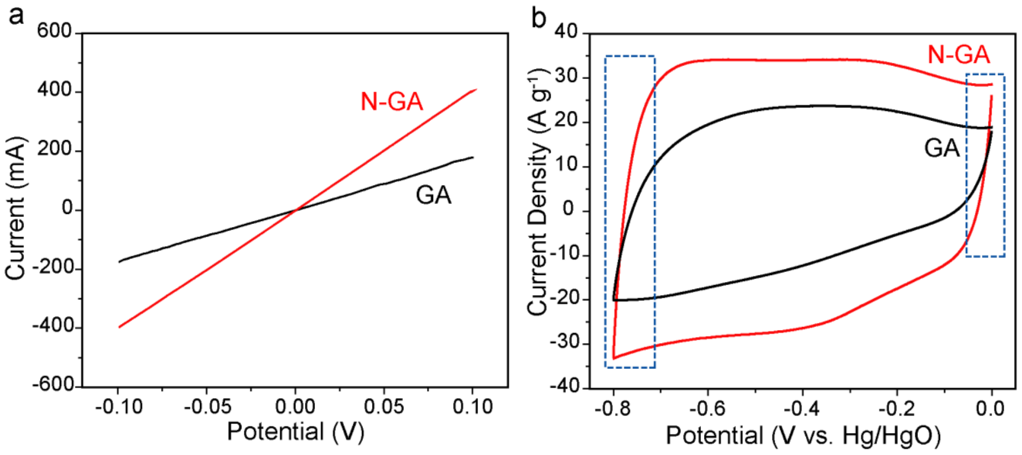

Figure S7. (a) *I-V* curve collected for GA and N-GA in the absence of electrolyte using a two-electrode system with an electrochemical workstation. The distance between two probes is fixed to be 1.0 cm. (b) CV diagrams of GA and N-GA collected in 3 M KOH aqueous electrolyte at a scan rate of 100 mV/s. The blue dashed boxes highlight the region near the boundaries of the potential window where the two CV diagrams show different slopes.

Figure S7a shows the current (*I*) *vs.* potential (*V*) curves collected for N-GA and GA. The slope of the line is directly proportional to electrical conductivity. Obviously, N-GA exhibits a considerably higher slope than GA, indicating N-GA is more electrically conductive than GA. Figure S7b shows the cyclic voltammograms collected in a 3 M KOH aqueous electrolyte. It has been reported that the slope of cyclic voltammograms near two ends (highlighted by the two dashed boxes) is directly related to the electrical conductivity.[6] N-GA has a steeper slope than GA, suggesting a higher electrical conductivity.

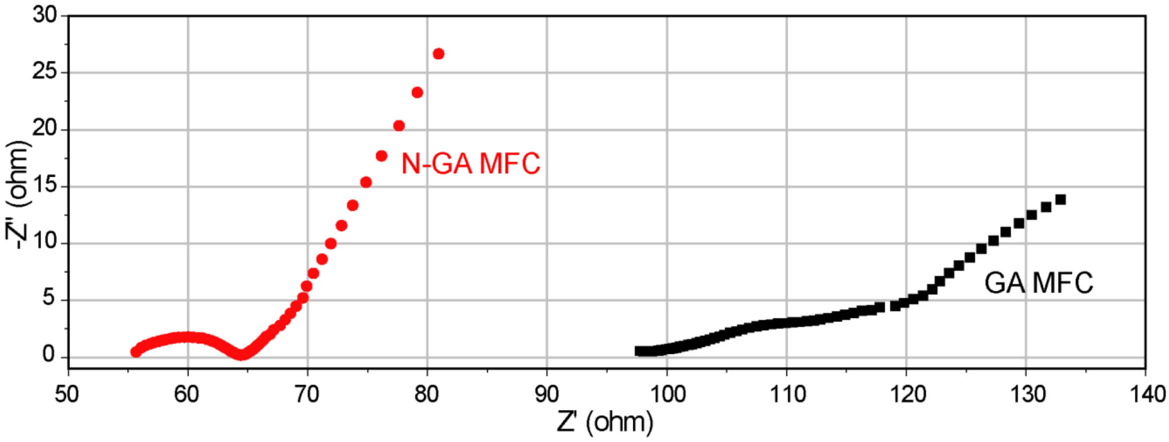

Figure S8. Nyquist plots collected for GA MFC and N-GA MFC in a frequency range from 0.01 Hz to 10000 Hz at the open circuit potential with a perturbation of 5 mV. The ohmic resistance is estimated from the Z'-intercept of the spectrum.

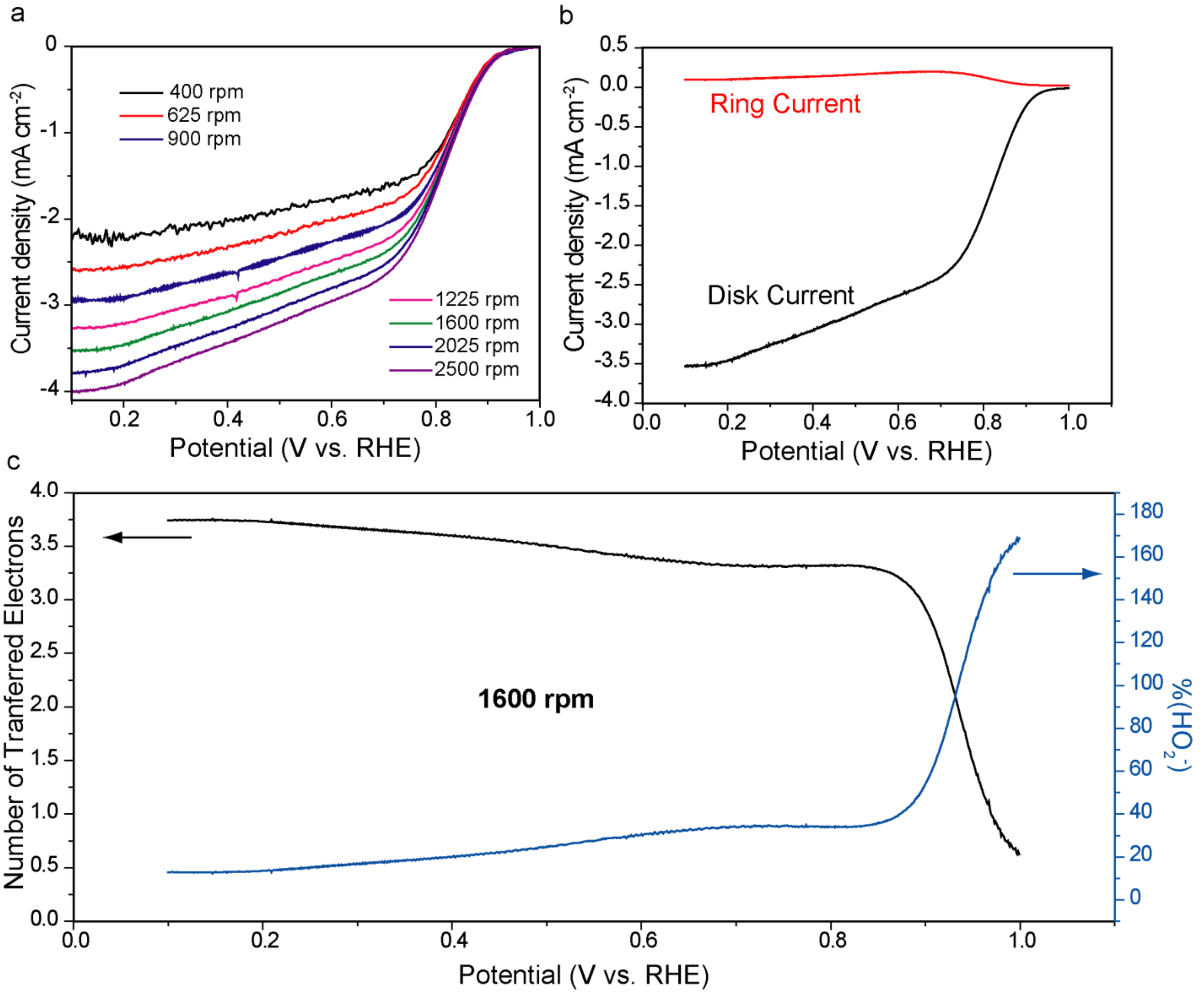

Figure S9. (a) RRDE curves of N-GA collected in O_2-saturated 0.1 M aqueous KOH solution at various rotation speeds. (b) Disk and ring current *vs.* potential curves collected at 1600 rpm in O_2-saturated 0.1 M aqueous KOH solution. (c) Number of transferred electrons (black curve) and yield of peroxide anion (blue curve) derived from the disc and ring current as a function of potential at 1600 rpm.

The rotating ring disk electrode (RRDE) measurement was performed to evaluate N-GA's oxygen reduction reaction (ORR) activity as a MFC cathode. Figure S9a shows the RRDE voltammograms collected at various scan rates. Cathodic currents at all scan rates started to emerge at approximately +0.88 V (*vs.* reversible hydrogen electrode, RHE). This on-set potential is only slightly lower than that of 10 wt% Pt/C (+0.94 *vs.* RHE).[7] The ring current is about 10 times lower than the disk current (Figure S9b), indicating peroxide species are rarely generated in the process. The number of transferred electrons (n) can be estimated based on the following equation: [8]

$$n = \frac{4I_D}{(I_D+\frac{I_R}{N})}$$

where I_R is the ring current, I_D the disk current and N the collection efficiency (37%).[9] At the high

rotating speed (1600 rpm), *n* increases drastically from 0 to 3.45 in the potential range from 1.0 to 0.82 V (Figure S9c). The maximum total number of transferred electrons is 3.75, suggesting a four-electron pathway dominates the reduction process. The yield of peroxide anion [%(HO_2^-)] can be determined by the following equation:[10]

$$\%(HO_2^-) = 200 \times \frac{I_R/N}{(I_D+I_R/N)}$$

A low peroxide yield (13.3%) is obtained in the potential range of 0.1 V~0.2 V. It again verifies a four-electron pathway that leads to the formation of hydroxide ions is the major process. The catalytic performance of N-GA for ORR is comparable to some other carbon-based catalysts including carbon-supported Fe-N electrocatalyst,[11] and graphitized multi-walled carbon nanotubes.[12]

References

[1] (a) Y. Zhang, Z. Sun, H. Wang, Y. Wang, M. Liang, S. Xue, *RSC Adv.* **2015**, 5, 10430; (b) F. Zheng, Y. Yang, Q. Chen, *Nat. Commun.* **2014**, 5, 5261.

[2] G. y. Jarzabek, Z. Borkowska, *Electrochim. Acta.* **1997**, 42, 2915.

[3] S. Konopka, B. McDuffie, *Anal. Chem.* **1970**, 42, 1741.

[4] S. A. Hasan, E. K. Tsekoura, V. Sternhagen, M. Strømme, *J. Phys. Chem. C* **2012**, 116, 6530.

[5] S. Cheng, B. E. Logan, *Electrochem. Commu.* **2007**, 9, 492.

[6] Z. Bo, W. Zhu, W. Ma, Z. Wen, X. Shuai, J. Chen, J. Yan, Z. Wang, K. Cen, X. Feng, *Adv.Mater.* **2013**, 25, 5799.

[7] J. Lu, W. Zhou, L. Wang, J. Jia, Y. Ke, L. Yang, K. Zhou, X. Liu, Z. Tang, L. Li, *ACS Catal.* **2016**, 6, 1045.

[8] M. Lefèvre, J.-P. Dodelet, *Electrochim. Acta.* **2003**, 48, 2749.

[9] K. Liu, Y. Song, S. Chen, *Nanoscale* **2015**, 7, 1224.

[10] G. Wu, K. L. More, C. M. Johnston, P. Zelenay, *Science* **2011**, 332, 443.

[11] C. W. Bezerra, L. Zhang, K. Lee, H. Liu, J. Zhang, Z. Shi, A. L. Marques, E. P. Marques, S. Wu, J. Zhang, *Electrochim. Acta.* **2008**, 53, 7703.

[12] M. J. Larsen, E. M. Skou, *J. Power Sources* **2012**, 202, 35.
